# Oral Anticoagulant Use and Appropriateness in Elderly Patients with Atrial Fibrillation in Complex Clinical Conditions: ACONVENIENCE Study

**DOI:** 10.3390/jcm11247423

**Published:** 2022-12-14

**Authors:** Clara Bonanad, Francesc Formiga, Manuel Anguita, Roberto Petidier, Alejandra Gullón

**Affiliations:** 1 Servicio de Cardiología, Hospital Clínico Universitario de Valencia, 46010 Valencia, Spain; 2 Instituto de Investigación Sanitaria (INCLIVA), 46010 Valencia, Spain; 3 Departamento de Medicina, Universidad de Valencia, 46010 Valencia, Spain; 4 Servicio de Medicina Interna, Hospital Universitari de Bellvitge, 08907 L’Hospitalet de Llobregat, Barcelona, Spain; 5 Servicio de Cardiología, Hospital Universitario Reina Sofía, Instituto Maimonides de Investigación Biomedica (IMIBIC), Universidad de Cordoba, 14004 Cordoba, Spain; 6 Servicio de Geriatría, Hospital Universitario de Getafe, 28905 Getafe, Madrid, Spain; 7 Servicio de Medicina Interna, Hospital Universitario de La Princesa, 28006 Madrid, Spain

**Keywords:** atrial fibrillation, anticoagulants, geriatric assessment, frail elderly, drug interactions, stroke, bleeding, factor Xa inhibitors, antithrombins

## Abstract

Non-valvular atrial fibrillation (NVAF) is the most common arrhythmia in older patients. Although direct-acting oral anticoagulants (DOAC) are the antithrombotic treatment of choice, irrespective of age, certain factors may limit their use. The aim of the ACONVENIENCE study was to consult the opinion of a multidisciplinary panel of experts on the appropriateness of using OACs in elderly patients (>75 years) with NVAF associated with certain complex clinical conditions. A consensus project was performed on the basis of a systematic review of the literature, and application of a two-round Delphi survey. The agreement of 79 panellists on 30 Delphi-type statements was evaluated, and their opinion on the appropriateness of different oral anticoagulants in 16 complex clinical scenarios was assessed. A total of 27 consensus statements were agreed upon, including all statements addressing anticoagulation in older patients and in patients at high risk of bleeding complications, and most of those addressing frailty, dementia, risk of falling, and complex cardiac situations. It was almost unanimously agreed upon that advanced age should not influence the anticoagulation decision. Apixaban was the highest-rated therapeutic option in 14/16 situations, followed by edoxaban. There is a high degree of agreement on anticoagulation in older patients with NVAF. Age should not be the single limiting factor when prescribing OACs, and the decision should be made based on net clinical benefit and a comprehensive geriatric assessment. Apixaban, followed by edoxaban, was considered the most appropriate treatment in the various complex clinical situations examined.

## 1. Introduction

Atrial fibrillation (AF) is the most common type of cardiac arrhythmia and is estimated to affect more than one million people in Spain [1]. The prevalence of AF in older adults is high [1,2], since age is an independent risk factor for this condition [3,4]. In the general Spanish population, the prevalence of AF is 4.6% in individuals between 60 and 69 years; 9.3% between 70 and 79 years; and 17.7% after the age of 80. Advanced age is also an independent risk factor for stroke, systemic thromboembolism, and bleeding [5,6].

The therapeutic options available for the prevention of stroke in patients with non-valvular AF (NVAF) are vitamin K antagonists (VKAs) and direct-acting oral anticoagulants (DOACs). Current clinical practice guidelines recommend DOACs as the treatment of choice, regardless of age [7]. DOACs have also been reported to significantly reduce the risk of stroke and systemic thromboembolism in older patients, without increasing the risk of major bleeding (even more than in younger patients [8]) and have a more favourable risk–benefit profile than VKAs [7]. Despite these data, older patients are currently less likely to receive anticoagulant therapy [9,10,11,12] and, in real clinical practice, the risk of bleeding is given more weight than the risk of stroke in therapeutic decision-making.

The geriatric population is increasing worldwide as life expectancy improves [13], but advanced age is associated with a parallel rise in multimorbidity, polypharmacy, falls, frailty, and dementia [14]. All these factors imply that ageing and its associated problems must be addressed when deciding on the treatment of NVAF. To date, two documents have reviewed and evaluated the use of oral anticoagulation in older patients with NVAF in Spain [15,16]. However, there is still much uncertainty surrounding the aptness of anticoagulant treatment for older patients, and the therapeutic options in complex clinical scenarios commonly presented by these patients. The lack of randomised, comparative clinical studies among the different DOACs makes it difficult to select the most suitable option.

The ACONVENIENCE study aimed to consult the opinion of a panel of experts on the prevention of stroke with OACs (VKAs and DOACs) in elderly patients (≥75 years) with NVAF who present certain complex clinical conditions and comorbidities, through a Delphi-like methodology.

## 2. Materials and Methods

The ACONVENIENCE study is a consensus study based on a literature review, a summary of the available scientific evidence, and the use of a two-round Delphi-type survey.

A scientific committee was formed, consisting of five participants with experience in the management of NVAF in older patients, from three specialities involved in the care of these patients (cardiology, internal medicine, and geriatrics). The scientific committee was responsible for decision-making and developed the Delphi questionnaire based on the results of the systematic review and modified it before the second round. The results were analysed by the committee in a fruitful discussion.

A research protocol was developed that described the objectives and methodology of the project, and the criteria and requirements for the selection of survey respondents. The scientific committee validated the protocol and developed 18 clinical questions (Annex 1), following the PICO method (Patient, Intervention, Comparison, Outcomes) [17].

**Figure 1:**
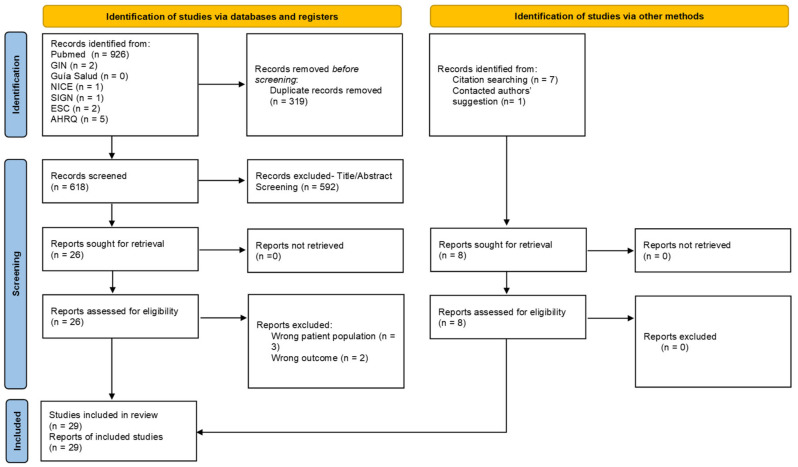
PRISMA 2020 flow diagram of the databases and registry search performed for the systematic literature review.

In July 2021, a rapid systematic review was conducted of the PubMed database, and the following clinical practice guideline repositories: Guidelines International Network (GIN), National Institute for Health and Care Excellence (NICE), Scottish Intercollegiate Guidelines Network (SIGN), European Society of Cardiology (ESC), Guía Salud, and Agency for Healthcare Research and Quality (AHRQ). Clinical practice guidelines, meta-analyses, systematic reviews, and consensus documents were prioritised, and only publications in English or Spanish were reviewed. If no data were found in these documents, then the search was extended to all kinds of publications. Overall, 945 publications were identified, of which 319 were duplicates. The title and abstract of 626 references were evaluated, according to the previously established inclusion and priority criteria, and 34 references were selected for a full reading. After the selected documents were read, five were discarded because they did not meet the inclusion criteria, and 29 publications were finally included in the synthesis of evidence (Figure 1).

To answer the clinical questions formulated, based on the synthesis of evidence and their expert judgement, the scientific committee agreed on 30 statements to be included in the Delphi-type questionnaire. The degree of agreement was assessed on a scale of 1 to 4 (1: strongly disagree, 2: moderately disagree, 3: moderately agree, 4: strongly agree). In 16 specific clinical scenarios addressed in certain statements, an additional question was included to assess the use of each of the available oral anticoagulants (apixaban, dabigatran, edoxaban, rivaroxaban and VKAs), on a scale of 1 to 10 (1: minimum score; 10: maximum score).

A panel of 100 participants was selected to form a representative series of hospitals and geographic areas who were not scientific committee members. Panellists had to meet the following criteria: (1) specialist in cardiology, internal medicine, geriatrics, haematology, or neurology; (2) experienced in the management of older patients with NVAF and DAOC treatment; (3) members of scientific society working groups; (4) seeing a reasonable number of older patients with NVAF per month; and (5) authors of related publications in indexed journals.

The consensus process was conducted using the Delphi two-round methodology [18]. The questionnaire was made available on an online platform that offered access to the summary of the evidence and space for panellists to include their comments.

In the interpretation of results, degrees of consensuses 1 and 2 were considered as disagreement, and 3 and 4 as agreement. Statements that obtained 100% agreement were accepted unanimously, and those with an agreement equal to or greater than 80% were accepted by consensus. Statements that obtained an agreement of between 79% and 66% were considered discrepant, and those that achieved an agreement of less than 66% were rejected. After the first round, the results and comments of the panellists were analysed, the relevant modifications were made, and the second round of the questionnaire was generated. Statements that reached consensus in the first round and those that did not require modification were not submitted to the second round. Questions assessing therapeutic options for different clinical situations were analysed by calculating the mean value.

## 3. Results

### 3.1. Participants

Of the 100 panellists invited, a total of 79 participated in both the first and second round (79% participation). Overall, 34.2% were specialists in internal medicine; 31.6% in cardiology; 17.7% in geriatrics; the percentage of specialists in neurology and haematology was less than 10%. Almost all panellists (97.5%) practised within the hospital setting, and 93.7% had more than ten years of experience in the care of older patients with NVAF. In total, 87% saw at least 25 patients per month (Table 1).

### 3.2. Delphi Questionnaire Results

In the first round, 29 Delphi statements were evaluated and 26 were agreed upon, 6 by unanimous agreement and 21 by consensus. The outcome of one of the statements was a discrepancy, while two were rejected. In the second round, after the appropriate modifications were made, 30 statements were evaluated and 27 achieved consensuses: 6 by unanimity and 20 by consensus. Three statements were judged to be discrepant, and none were rejected (Table 2). The details of the percentages of panellists voting on each of the scores [1,2,3,4] for the statements is shown in Table S1.

The following statements achieved unanimous agreement:

- The use of DOACs, rather than VKAs, is recommended for stroke prevention in older patients with NVAF.
- DOACs have a more favourable risk–benefit profile than VKAs in frail patients.
- Apixaban may have a more favourable risk–benefit profile than VKAs in patients at risk of falling.
- Anticoagulation should be avoided or administered with extreme caution in patients with fewer than 50,000 platelets/mL.
- High risk of bleeding should not automatically prompt the withdrawal of anticoagulation before monitoring modifiable bleeding risk factors and instigating closer patient follow-up.
- Polypharmacy requires greater awareness of drug interactions in patients with high bleeding risk.

Almost three-quarters (73%) of respondents agreed that the evidence is insufficient to specify a DOAC of choice in elderly patients with NVAF and low body weight (<60 kg), and that the dose should be reduced according to the Summary of Product Characteristics (SmPC), if necessary (Table 2). Differences in the degree of agreement among the different specialities were analysed and found to be 100% among haematology specialists and 86% among neurology specialists, while the other specialities did not reach consensus (Figure S1). However, haematology and neurology were the specialities with less representation in the study, therefore it limits the interpretation of this divergence.

No agreement was reached on long-term anticoagulation in patients with no previous AF who have acute coronary syndrome (ACS) and an isolated episode of peri-infarction AF (Table 2). This statement was not brought to the second round because of a lack of evidence, and at the discretion of the scientific committee. In this case, haematology specialists reached unanimity and geriatricians and neurologists reached consensus, but cardiologists and internists did not (Figure S1).

Consensus was not reached on the statement that, given the lack of randomised clinical trials with DOAC, VKAs are the gold standard in patients with intraventricular thrombus associated with acute myocardial infarction (AMI), but that the use of DOAC could be considered in very special situations (Table 2). Consensus was reached among haematology and internal medicine specialists, but not among neurologists, cardiologists, and geriatricians (Figure S1).

### 3.3. Results of the Evaluation of Therapeutic Options in the Clinical Scenarios under Consideration

**Figure 2:**
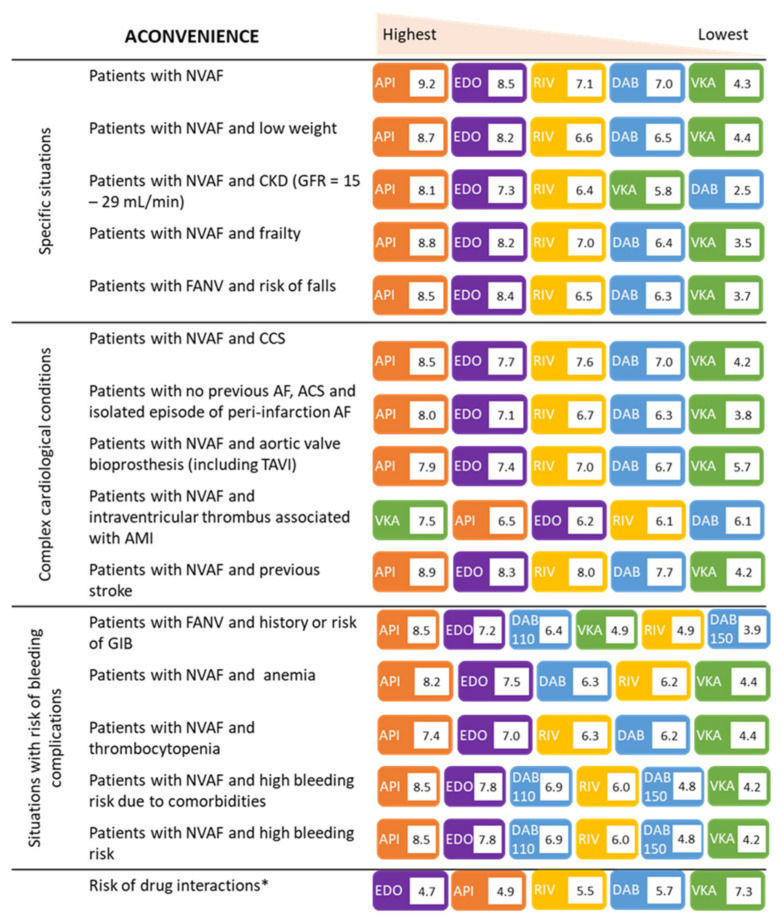
Mean score for available anticoagulation options in different clinical situations that may be encountered in elderly patients (maximum score = 10; minimum score = 1). ACS, acute coronary syndrome; AMI, acute myocardial infarction; API, apixaban; CCS, chronic coronary syndrome; CKD, chronic kidney disease; DAB, dabigatran; EDO, edoxaban; GFR, glomerular filtration rate; GIB, gastrointestinal bleeding; NVAF, non-valvular atrial fibrillation; RIV, rivaroxaban; TAVI, transcatheter aortic valve implantation; VKA, vitamin K antagonists. * The question was made on the OAC with less risk of drug interactions.

Apixaban was the highest-rated drug in 14 of the 16 clinical situations in older patients with NVAF and obtained a mean score of ≥8 in 12. Edoxaban was the second highest-rated drug in 14 of the 16 proposed clinical situations. Rivaroxaban was the third option in ten clinical situations, the fourth option in four, and the fifth in one. Dabigatran was always considered the fourth or fifth option, except for the 110 mg dose, which was the third option in patients with high bleeding risk because of their general status or comorbidities. VKAs were the worst-rated therapeutic option in 13 of the 16 clinical situations and obtained a mean score of <5 in 11 (Figure 2).

In older patients with NVAF in general and, specifically, in those with low weight, chronic kidney disease, frailty, and risk of falling, apixaban was the option most often selected, followed by edoxaban.

In all complex cardiological scenarios that were proposed [chronic coronary syndrome (CCS), no previous NVAF, and ACS with isolated episode of peri-infarction NVAF, aortic bioprosthesis, and previous stroke], apixaban, followed by edoxaban, was considered the most appropriate option, except in patients with intraventricular thrombus associated with AMI. In these patients, panellists scored VKAs as the first option, while the second choice was apixaban.

In all the bleeding risk situations that were proposed [risk or history of gastrointestinal bleeding (GIB)], anaemia, thrombocytopenia, high bleeding risk due to comorbidities or other reasons, apixaban scored the highest, followed by edoxaban (Figure 2).

In assessing the risk of drug interactions in older patients with NVAF, edoxaban was the therapeutic option that scored highest, followed by apixaban (Figure 2).

## 4. Discussion

The results obtained in this study show a high degree of agreement regarding anticoagulation in older patients with NVAF among clinicians caring for these patients, even with the participation of five different specialities. The panel of participants agreed on 90% and unanimously accepted 20% of the proposed Delphi statements.

Anticoagulant treatment for the prevention of stroke is a challenge in the elderly population in particular, since it is a heterogeneous group with greater comorbidity, polypharmacy, frailty, and cognitive impairment, along with functional and psychosocial issues [19,20], and increased thromboembolic and bleeding risks [5,6]. Age has been identified as one of the barriers to physicians prescribing anticoagulants [21]. However, current European clinical practice guidelines recommend that stroke risk should be assessed using the CHA2DS2-VASc score, and that age should not be considered as the sole factor when considering anticoagulant therapy [7,22]. In line with this recommendation, panellists agreed that advanced age, per se, should not influence the anticoagulation decision. The guidelines also recommend DOACs as a preferred option for anticoagulation in older patients [7,22], since they confer a reduction in the risk of stroke without increasing the risk of major bleeds in this population; therefore, they offer a greater net clinical benefit (the composite variable that includes the risk of thromboembolic and bleeding events) than VKAs [8,23,24]. The study participants unanimously accepted this recommendation, and also agreed that advanced age should not be a single criterion when prescribing reduced doses of DOACs. Nevertheless, undertreatment and underdosing remain as issues in the anticoagulant treatment of older patients [25,26,27], and the consequences of inappropriate DOAC dosing include an increased risk of stroke, and bleeding [28,29]. It has also been shown that the net clinical benefit is greater in the elderly population receiving standard doses, than in those treated at low doses [30]. To avoid the age barrier and ensure safe administration and dosing of DOACs, a comprehensive geriatric assessment must be performed, including life expectancy, cognitive performance, functional status and comorbidities, to determine the net clinical benefit based on the specific patient situation.

Weight and body mass index are important variables for drug distribution and plasma concentration levels. Neither one of these factors was an exclusion criterion in randomised trials with DOACs, although dose reductions for low body weight (≤60 kg) were mandatory for both apixaban and edoxaban in patients ≥80 years and/or creatinine ≥1.5 mg/dL [31,32,33,34]. DOACs are effective and safe in patients at either end of the weight spectrum (high or low) [35]. However, the lack of specific randomised clinical trials in this population with each of the DOACs may be the cause of the discrepancies observed in this study, especially among specialists in geriatrics, internal medicine, and cardiology. The specific dose reductions available for apixaban and edoxaban [32,34] are the reason their uses are recommended as preferred options [16,36].

Frailty, cognitive impairment, and risk of falls should also not be reasons for avoiding anticoagulant treatment [7,36], except in cases of severe frailty or very limited life expectancy. This was also the perception of most study participants. However, the evidence shows that frail patients are less likely to be receiving anticoagulant treatment at the time of hospitalisation than those who are not frail [37]. Early identification of frailty is important, since it may be at least partially reversible. There are numerous scales designed for this purpose, but the lack of a single definition and universally accepted validated scale makes diagnosis more difficult [38].

Anticoagulation may have an added protective role in patients by reducing the risk of cognitive impairment [39]. Some studies also indicate that DOACs are superior to VKAs in this regard [40] and that, moreover, the limited time within the therapeutic range is associated with dementia, in patients treated with VKAs [41,42]. Falls, furthermore, should not be a contraindication to the use of DOACs, although it is important to take precautions and evaluate risk factors for bleeding that can be modified [36]. Apixaban, for example, has shown superior efficacy and safety compared with warfarin, irrespective of a history of falls [43]. Edoxaban, for its part, has been associated with a greater reduction in severe bleeding events and mortality in patients at risk of falling [44].

Complex cardiac conditions in older patients with NVAF that reached consensus among a large majority of the panel of experts are reflected in the current guidelines [7,36]. This is the case for patients with CCS, who must remain on monotherapy for 12 months after the acute event, preferably with a DOAC. This is also the case for patients with aortic valve bioprostheses, in whom DOACs can be used as an alternative to VKAs. Indeed, there is no evidence that anticoagulant therapy must be modified in the presence of valvular heart disease, except for in severe moderate mitral stenosis or a mechanical heart valve [7]. Finally, as the same guidelines state, the preferred option in patients with previous stroke is a DOAC, since the risk of ischemic events is higher in patients treated with VKAs [45]. In contrast, no consensus was reached on the use of DOACs in patients with intraventricular thrombus associated with AMI, especially among specialists in neurology, cardiology, and geriatrics. Although some studies have been published in patients treated with DOACs [46,47,48,49,50,51], there is still no solid evidence on this topic, and in this clinical situation VKAs remain the current gold standard of treatment. The guidelines indicate that in special situations (no VKA monitoring available, unstable INR, etc.), DOACs may be an option, provided the patient is informed of the lack of evidence, and gives their consent for off-label use [36].

Regarding older patients with NVAF at risk of bleeding complications, the respondents agreed with all the proposed statements. Until some years ago, the lack of availability of antidotes could be a limiting factor for DOAC prescription in patients with high risk of bleeding. However, nowadays, having the specific reversal agents idarucizumab and andexanet alfa, confers a higher degree of safety to the prescribers, and can increase patient and physician acceptance of DOAC treatment [52,53]. According to current guidelines, the administration of apixaban or dabigatran 110 mg should be considered in patients who have experienced a recent bleeding event, since these options are not associated with a higher risk of GIB than warfarin [7]. Age has not been shown to increase the risk of major bleeds with DOACs, compared with VKAs, except in the case of high-dose dabigatran [53,54]. Indeed, apixaban and edoxaban appear to reduce the risk of major bleeding, even in older groups [32,55,56].

The net clinical benefit of anticoagulation in NVAF is favourable to DOACs, both in the general population and in older patients [57,58,59,60] and is maintained longer with DOACs than with VKAs [30,61]. A recent meta-analysis has also demonstrated that DOACs are more effective compared to warfarin in octogenarians with AF [62]. For this reason, VKAs were the least valued treatment option in almost all clinical situations proposed in this study, except in patients with intraventricular thrombus associated with AMI, for which no evidence or indication is available for DOACs.

Despite the robust evidence for some of the proposed scenarios, and panellists expressing their preference for DOACs, the reality is that most older patients continue to receive VKAs [20]. The lack of comparative studies on the different DOACs makes it difficult to decide which one is most suitable for an older population. The patients included in the various DOAC pivotal studies had different baseline characteristics, which makes a direct comparison impossible. In this study, apixaban and edoxaban, first and second, respectively, were the DOACs considered most suitable in general, and in complex situations such as low weight, chronic kidney disease, frailty, risk of falls, and even in patients with certain cardiovascular or bleeding complications.

The design of this study has some limitations inherent to the chosen methodology. Although it was conducted using a robust, well-known, rigorous methodology, based on the Delphi technique, it only provides us with qualitative information on the degree of agreement among the panellists, based on the available evidence, and their clinical practice and experience. Furthermore, since this is a Delphi project, the possibility exists that each respondent might interpret the proposed statements differently; to counteract this limitation, the statements were modified before the second round, in line with the comments of the panellists who had responded to the first round.

## 5. Conclusions

There is a high degree of agreement on anticoagulant therapy in older patients with NVAF. There is a broad consensus that age, per se, should not be a decisive factor in prescribing anticoagulation in these patients. It was also widely agreed that the decision should be made based on the net clinical benefit, and a comprehensive geriatric assessment of each patient. Older patients have shown greater net benefit with DOACs than with VKAs, and DOACs should, therefore, be the preferred option. Apixaban is considered the most appropriate DOAC in the various complex clinical situations examined in this study. Edoxaban was the second option in these situations.

## Figures and Tables

**Table 1:** Panellist characteristics (*n* = 79).

Characteristics	*n* (%)
Specialty	
Internal medicine	27 (34.2)
Cardiology	25 (31.6)
Geriatrics	14 (17.7)
Neurology	7 (8.9)
Haematology	6 (7.6)
Workplace	
Private consultation	1 (1.3)
Consultation in specialised centre	1 (1.3)
Hospital	77 (97.5)
Experience in the management of elderly patients (>75 years) with NVAF	
5–9 years	5 (6.3)
10–14 years	17 (21.5)
15–19 years	18 (22.8)
20–24 years	15 (19.0)
25–29 years	13 (16.5)
30–35 years	11 (13.9)
Patients seen every month	
<25	(13)
25–50	(50)
>50	(37)

**Table 2:** Percentage of agreement on Delphi-type statements on oral anticoagulation in elderly patients (>75 years) with NVAF.

No.	STATEMENT	% Agreement	Round
**Block 1. Anticoagulation in the elderly patient with NVAF**			
1	In patients with NVAF, advanced age per se should not influence the anticoagulation decision.	99%	2nd
2	In line with current ESC guidelines, the use of DOACs, rather than VKAs, is recommended for the prevention of stroke in older patients with NVAF (except for patients with mechanical valves or moderate to severe mitral stenosis).	100%	1st
3	Advanced age should not be the only criterion for avoiding the full dose of anticoagulation in patients with NVAF.	99%	1st
**Block 2. Impact of body weight, drug interactions, and renal function on oral anticoagulation in older patients with NVAF**			
4	There is insufficient evidence to identify a DOAC of choice for use in elderly NVAF patients with low body weight (<60 kg). The dose will be adjusted, if necessary, according to the dose reduction criteria specified in the SmPC.	73%	2nd
5	Interactions that may be decisive for the choice of DOAC in older patients with NVAF are: - potent P-gp and CYP3A4 inhibitors - strong P-gp and/or CYP3A4 inducers.	97%	1st
6	The data currently available on the use of DOAC in patients with CKD with creatinine clearance 15–30 mL/min are limited by the exclusion of these patients from clinical trials, so the compound with the greatest net clinical benefit and least disease progression cannot be identified.	94%	2nd
7	Rivaroxaban, edoxaban or apixaban, in adjusted doses, are a viable option for severe CKD (CrCl 15–30 mL/min). The use of dabigatran is contraindicated in these patients.	99%	2nd
**Block 3. Impact of frailty, dementia, and risk of falling on oral anticoagulation in older patients with NVAF**			
8	In older patients with NVAF, frailty without a disability should not be a determinant for avoiding anticoagulants in terms of net clinical benefit.	99%	2nd
9	DOACs have a more favourable risk-benefit profile than VKAs in frail older patients with NVAF.	100%	1st
10	Cognitive impairment should not generally be a reason to avoid anticoagulation in older patients with NVAF.	85%	1st
11	Avoiding anticoagulation is an option in older patients with NVAF and advanced dementia, provided the patient’s primary caregiver agrees.	96%	2nd
12	Apixaban may have a more favourable risk-benefit profile than VKAs in older patients with NVAF and risk of falls.	100%	2nd
13	Edoxaban may have a more favourable risk-benefit profile than VKAs in older patients with NVAF and risk of falls.	95%	2nd
**Block 4. Impact of complex cardiological conditions on oral anticoagulation in older patients with NVAF**			
14	In older patients with CCS and NVAF, antiplatelet therapy should be withdrawn 12 months after the acute event and/or coronary revascularisation, and only an anticoagulant should be continued, preferably a DOAC.	97%	1st
15	Older patients without previous NVAF who have ACS and develop an isolated episode of peri-infarction NVAF should receive long-term anticoagulation.	77%	1st
16	In older patients with NVAF and aortic valve bioprosthesis, including TAVI, the use of DOACs is a plausible alternative to VKAs.	95%	1st
17	In older patients with NVAF and intraventricular thrombus associated with AMI, the gold standard is VKA, due to the lack of randomised clinical trials with DOAC. However, despite this lack of evidence, the use of DOAC could be considered in very special situations.	70%	2nd
18	In older patients with NVAF and previous stroke, a DOAC should be preferred over a VKA.	97%	1st
**Block 5. Impact of a high risk of bleeding complications on oral anticoagulation in older patients with NVAF**			
19	The use of DOAC may be associated with an increased risk of GIB compared with VKA. In older patients with NVAF and history or high risk of GIB who are candidates for DOAC treatment, the use of apixaban or dabigatran 110 mg is recommended, as a risk of GIB similar to that of warfarin has been demonstrated.	91%	1st
20	Treatment and correction of reversible causes and risk factors are key to minimising GIB. In patients with NVAF, the use of PPI combined with anticoagulation therapy is recommended to minimise the risk of GIB, especially in patients with a history of bleeding and/or ulcers.	99%	1st
21	Moderate-severe anaemia (Hb < 11 g/dL) is associated with an increased risk of bleeding complications in patients with NVAF receiving anticoagulation. However, it has not been associated with reduced antithrombotic efficacy.	92%	1st
22	All reversible causes of anaemia and predisposing causes (including drugs) that could increase the risk of bleeding before and during anticoagulant treatments should be investigated.	99%	1st
23	The use of anticoagulation in older patients with NVAF and thrombocytopenia should be performed by a multidisciplinary team, on an individualised basis, balancing the patient’s thrombotic and bleeding risk and correcting all reversible causes.	99%	1st
24	Anticoagulation should be avoided or used with extreme caution in patients with platelet counts below 50,000 platelets/mL.	100%	1st
25	DOACs appear to have a better safety and efficacy profile than VKA in patients with NVAF and thrombocytopenia.	95%	1st
26	A high bleeding risk due to comorbidities in older patients with NVAF is not an absolute contraindication to the use of oral anticoagulants. An individualised approach is essential.	97%	1st
27	A high bleeding risk should not automatically lead to the withdrawal of anticoagulants in older patients with NVAF and risk of stroke. In these patients, monitoring of all modifiable bleeding risk factors and close follow-up are essential.	100%	1st
28	Polypharmacy requires us to be more alert to drug interactions.	100%	1st
29	In older patients with NVAF and high bleeding risk, treatment with DOAC has been associated with a similar or lower risk of major bleeding, compared to VKA.	94%	1st
30	All DOACs are associated with a reduced risk of ICH compared with VKAs.	90%	1st

ACS, acute coronary syndrome; AMI, acute myocardial infarction; CCS, chronic coronary syndrome; CKD, chronic kidney disease; CrCl, creatinine clearance (Cockcroft–Gault equation); DOAC, direct-acting oral anticoagulant; ESC, European Society of Cardiology; GIB, gastrointestinal bleeding; ICH, intracranial haemorrhage; NVAF, non-valvular atrial fibrillation; PPI, proton pump inhibitors; TAVI, transcatheter aortic valve implantation; VKA, vitamin K antagonist.

## Supplementary Materials

The following supporting information can be downloaded at: https://www.mdpi.com/article/10.3390/jcm11247423/s1, Figure S1: Percentage of agreement on Delphi-type questionnaire statements on oral anticoagulation in elderly patients (>75 years) with NVAF that did not reach consensus. Table S1: Detailed panelist responses.
