# Supplementary material for: Oral Anticoagulant Use and Appropriateness in Elderly Patients with Atrial Fibrillation in Complex Clinical Conditions: ACONVENIENCE Study
Source: J Clin Med. 2022 Dec 14;11(24):7423. doi: 10.3390/jcm11247423 (PMC9781896; doi:10.3390/jcm11247423)
Supplement: Supplementary file 1 [file jcm-11-07423-s001.zip › Supp. Table S1_Detailed panelists responsesAG.pdf]

Oral anticoagulant use and appropriateness in elderly patients with atrial fibrillation in complex clinical conditions: ACONVENIENCE study. Delphi results (two rounds)

	Number of panelists in agreement or disagreement with the statement				Percentage of panelists in agreement or disagreement with the statement				Percentage of panelists in agreement or disagreement with the statement				Round	Results
	Agreement		Disagreement		Agreement		Disagreement		Agreement (4+3)		Disagreement (2+1)			
Statement	4	3	2	1	4	3	2	1	n	%	n	%		
Block 1. Anticoagulation in the elderly patient with NVAF														
**In patients with NVAF, advanced age per se should not influence the anticoagulation decision.**	73	5	1	0	92%	6%	1%	0%	78	**99%**	1	1%	2	**Consensus**
**In line with current ESC guidelines, the use of DOACs, rather than VKAs, is recommended for the prevention of stroke in older patients with NVAF (except for patients with mechanical valves or moderate to severe mitral stenosis).**	74	5	0	0	94%	6%	0%	0%	79	**100%**	0	0%	1	**Unanimity**
**Advanced age should not be the only criterion for avoiding the full dose of anticoagulation in patients with NVAF.**	71	7	1	0	90%	9%	1%	0%	78	**99%**	1	1%	1	**Consensus**

**Impact of body weight, drug interactions, and renal function on oral anticoagulation in older patients with NVAF**														
**There is insufficient evidence to identify a DOAC of choice for use in elderly NVAF patients with low body weight (< 60kg). The dose will be adjusted, if nec-essary, according to the dose reduction criteria specified in the SmPC.**	19	39	18	3	24%	49%	23%	4%	58	**73%**	21	27%	2	**Discrepancy**
**Interactions that may be decisive for the choice of DOAC in older patients with NVAF are:<br>- potent P-gp and CYP3A4 inhibitors<br>- strong P-gp and/or CYP3A4 inducers.**	49	28	1	1	62%	35%	1%	1%	77	**97%**	2	3%	1	**Consensus**
**The data currently available on the use of DOAC in patients with CKD with creatinine clearance 15-30 mL/min are limited by the exclusion of these patients from clinical trials, so the compound with the greatest net clinical benefit and least disease progression cannot be identified.**	42	32	4	1	53%	41%	5%	1%	74	**94%**	5	6%	2	**Consensus**
**Rivaroxaban, edoxaban or apixaban, in adjusted doses, are a viable option for severe CKD (CrCl 15-30 mL/min). The use of dabigatran is contraindicated in these patients.**	61	17	1	0	77%	22%	1%	0%	78	**99%**	1	1%	2	**Consensus**
**Block 3. Impact of frailty, dementia, and risk of falling on oral anticoagulation in older patients with NVAF**														

**In older patients with NVAF, frailty without a disability should not be a determinant for avoiding anticoagulants in terms of net clinical benefit.**	61	17	1	0	77%	22%	1%	0%	78	**99%**	1	1%	2	**Consensus**
**DOACs have a more favourable risk-benefit profile than VKAs in frail older patients with NVAF.**	65	14	0	0	82%	18%	0%	0%	79	**100%**	0	0%	1	**Unanimity**
**Cognitive impairment should not generally be a reason to avoid anticoagulation in older patients with NVAF.**	39	28	8	4	49%	35%	10%	5%	67	**85%**	12	15%	1	**Consensus**
**Avoiding anticoagulation is an option in older patients with NVAF and advanced dementia, provided the patient's primary caregiver agrees.**	66	10	3	0	84%	13%	4%	0%	76	**96%**	3	4%	2	**Consensus**
**Apixaban may have a more favourable risk-benefit profile than VKAs in older patients with NVAF and risk of falls.**	63	16	0	0	80%	20%	0%	0%	79	**100%**	0	0%	2	**Unanimity**
**Edoxaban may have a more favourable risk-benefit profile than VKAs in older patients with NVAF and risk of falls.**	53	22	4	0	67%	28%	5%	0%	75	**95%**	4	5%	2	**Consensus**

Block 4. Impact of complex cardiological conditions on oral anticoagulation in older patients with NVAF

**In older patients with CCS and NVAF, antiplatelet therapy should be withdrawn 12 months after the acute event and/or coronary revascularisation, and only an anticoagulant should be continued, preferably a DOAC.**	62	15	2	0	78%	19%	3%	0%	77	**97%**	2	3%	1	**Consensus**
**Older patients without previous NVAF who have ACS and develop an isolated episode of peri-infarction NVAF should receive long-term anticoagulation.**	29	32	14	4	37%	41%	18%	5%	61	**77%**	18	23%	1	**Discrepancy**
**In older patients with NVAF and aortic valve bioprosthesis, including TAVI, the use of DOACs is a plausible alternative to VKAs.**	43	32	3	1	54%	41%	4%	1%	75	**95%**	4	5%	1	**Consensus**
**In older patients with NVAF and intraventricular thrombus associated with AMI, the gold standard is VKA, due to the lack of randomised clinical trials with DO-AC. However, despite this lack of evidence, the use of DOAC could be considered in very special situations.**	14	41	23	1	18%	52%	29%	1%	55	**70%**	24	30%	2	**Discrepancy**
**In older patients with NVAF and previous stroke, a DOAC should be preferred over a VKA.**	66	11	2	0	84%	14%	3%	0%	77	**97%**	2	3%	1	**Consensus**
**Block 5. Impact of a high risk of bleeding complications on oral anticoagulation in older patients with NVAF**														

**The use of DOAC may be associated with an increased risk of GIB compared with VKA. In older patients with NVAF and history or high risk of GIB who are candidates for DOAC treatment, the use of apixaban or dabigatran 110 mg is recommended, as a risk of GIB similar to that of warfarin has been demonstrated.**	39	33	4	3	49%	42%	5%	4%	72	**91%**	7	9%	1	**Consensus**
**Treatment and correction of reversible causes and risk factors are key to mini-mising GIB. In patients with NVAF, the use of PPI combined with anticoagula-tion therapy is recommended to minimise the risk of GIB, especially in patients with a history of bleeding and/or ulcers.**	64	14	1	0	81%	18%	1%	0%	78	**99%**	1	1%	1	**Consensus**
**Moderate-severe anaemia (Hb < 11 g/dL) is associated with an increased risk of bleeding complications in patients with NVAF receiving anticoagulation. However, it has not been associated with reduced antithrombotic efficacy.**	38	35	5	1	48%	44%	6%	1%	73	**92%**	6	8%	1	**Consensus**
**All reversible causes of anaemia and predisposing causes (including drugs) that could increase the risk of bleeding before and during anticoagulant treatments should be investigated.**	72	6	1	0	91%	8%	1%	0%	78	**99%**	1	1%	1	**Consensus**
**The use of anticoagulation in older patients with NVAF and thrombocytopenia should be performed by a multidisciplinary team, on an individualised basis, balancing the patient's thrombotic and bleeding risk and correcting all reversible causes.**	64	14	1	0	81%	18%	1%	0%	78	**99%**	1	1%	1	**Consensus**

**Anticoagulation should be avoided or used with extreme caution in patients with platelet counts below 50,000 platelets/mL.**	64	15	0	0	81%	19%	0%	0%	79	**100%**	0	0%	1	**Unanimity**
**DOACs appear to have a better safety and efficacy profile than VKA in patients with NVAF and thrombocytopenia.**	34	41	4	0	43%	52%	5%	0%	75	**95%**	4	5%	1	**Consensus**
**A high bleeding risk due to comorbidities in older patients with NVAF is not an absolute contraindication to the use of oral anticoagulants. An individualised approach is essential.**	69	8	2	0	87%	10%	3%	0%	77	**97%**	2	3%	1	**Consensus**
**A high bleeding risk should not automatically lead to the withdrawal of antico-agulants in older patients with NVAF and risk of stroke. In these patients, moni-toring of all modifiable bleeding risk factors and close follow-up are essential.**	65	14	0	0	82%	18%	0%	0%	79	**100%**	0	0%	1	**Unanimity**
**Polypharmacy requires us to be more alert to drug interactions.**	74	5	0	0	94%	6%	0%	0%	79	**100%**	0	0%	1	**Unanimity**
**In older patients with NVAF and high bleeding risk, treatment with DOAC has been associated with a similar or lower risk of major bleeding, compared to VKA.**	50	24	4	1	63%	30%	5%	1%	74	**94%**	5	6%	1	**Consensus**

**All DOACs are associated with a reduced risk of ICH compared with VKAs.**	62	9	7	1	78%	11%	9%	1%	71	**90%**	8	10%	1	**Consensus**

Statements that obtained 100% agreement were accepted unanimously and those with an agreement equal to or greater than 80% were accepted by consensus. Statements that obtained an agreement of between 79% and 66% were considered discrepant, and those that achieved an agreement of less than 66% were rejected. ACS, acute coronary syndrome; AMI, acute myocardial infarction; CCS, chronic coronary syndrome; CKD, chronic kidney disease; CrCl, creatinine clearance (Cockcroft–Gault equation); DOAC, direct-acting oral anticoagulant; ESC, European Society of Cardiology; GIB, gastrointestinal bleeding; ICH, intracranial haemorrhage; NVAF, non-valvular atrial fibrillation; PPI, proton pump inhibitors; TAVI, transcatheter aortic valve implantation; VKA, vitamin K antagonist.
