# Supplementary material for: Oral Anticoagulant Use and Appropriateness in Elderly Patients with Atrial Fibrillation in Complex Clinical Conditions: ACONVENIENCE Study
Source: J Clin Med. 2022 Dec 14;11(24):7423. doi: 10.3390/jcm11247423 (PMC9781896; doi:10.3390/jcm11247423)
Supplement: Supplementary file 1 [file jcm-11-07423-s001.zip › Supplementary Figure S1_v1.0.pdf]

Supplementary Materials

Figure S1: Percentage of agreement on Delphi-type questionnaire statements on oral anticoagulation in elderly patients (>75 years) with NVAF that did not reach consensus.

	Global	Hematology	Neurology	Geriatrics	Internal medicine	Cardiology
4. There is insufficient evidence to identify a DOAC of choice for use in elderly NVAF patients with low body weight (< 60kg). The dose will be adjusted, if necessary, according to the dose reduction criteria specified in the SmPC.	73%	100%	86%	79%	81%	64%
15. Older patients without previous NVAF who have ACS and develop an isolated episode of peri-infarction NVAF should receive long-term anticoagulation.	77%	100%	86%	93%	63%	76%
17. In older patients with NVAF and intraventricular thrombus associated with AMI, the gold standard is VKA, due to the lack of randomised clinical trials with DOAC. However, despite this lack of evidence, the use of DOAC could be considered in very special situations.	70%	83%	71%	50%	70%	64%

DOAC, direct-acting oral anticoagulant; NVAF, non-valvular atrial fibrillation; ACS, acute coronary síndrome; AMI, acute myocardial infarction; VKA, vitamin K antagonists.
